# Presentation of Rectal Cancer Among Young Adults in a Tertiary Care Center

**DOI:** 10.7759/cureus.91992

**Published:** 2025-09-10

**Authors:** Amna Fareed, Mazhar Iqbal, Syed Shafqatullah, Naeem Khan, Resham Ali, Raja Jawad, Ali Muhammad Rahuja, Sarkhail A Sayar

**Affiliations:** 1 General and Colorectal Surgery, Jinnah Postgraduate Medical Centre, Karachi, PAK

**Keywords:** early detection of cancer, neoplasm staging, rectal neoplasms, signet ring cell carcinoma, young adult

## Abstract

Background: Rectal cancer, once predominantly associated with older adults, has shown a rising incidence among young adults in recent decades. This trend raises significant concerns about diagnostic delays, advanced disease presentation, and the unique challenges faced by this population. This study investigates the patterns and characteristics of rectal cancer among young adults presenting to a tertiary care center over a six-month period.

Methodology: A cross-sectional study was conducted, reviewing patients aged 18-45 years diagnosed with rectal cancer between January 2025 and June 2025. Data were collected on demographics, clinical presentation, tumor staging, histopathology, and treatment modalities. Statistical analyses were performed to identify trends and correlations, particularly focusing on symptomatology and delays in diagnosis.

Results: A total of 73 young adults (n = 73) were included, with a mean age of 32.5 ± 7.2 years. There was a male predominance, with 44 patients (60.3%) being male. The most common presenting symptom was per rectal bleeding, reported in 54 patients (74.0%), followed by altered bowel habits with lower abdominal pain in 10 patients (13.7%). Delayed diagnosis was common, as 53 patients (72.6%) presented at advanced stages (stage IIIB or IV). The lower rectum was the most frequent tumor site, observed in 35 patients (47.9%). Adenocarcinoma was the predominant histological subtype in 41 patients (56.2%), with a high prevalence of poorly differentiated tumors in 47 patients (64.4%) and signet ring cell carcinoma in 22 patients (30.1%). Common associated risk factors included a low-fiber diet in 42 patients (57.5%) and smoking in 18 patients (24.7%).

Conclusion: The rising incidence of rectal cancer in young adults and its tendency to present at advanced stages highlight the critical need for increased awareness among both clinicians and the public. Early recognition of symptoms and timely diagnostic evaluations are essential to improving outcomes. Targeted strategies, including awareness campaigns and risk stratification in younger populations, are recommended to address this emerging healthcare challenge.

## Introduction

The incidence of colorectal cancer (CRC) among individuals under the age of 50 years has been steadily increasing in both the United States and Europe over the past few decades, with the sharpest rise observed in rectal cancer (RC). An annual increase of up to 3.2% has been reported among the youngest age group (20-29 years) [1]. Although there is evidence of worse outcomes for younger patients with colon cancer, data regarding rectal cancer outcomes in this population remain limited [2]. The median age at diagnosis for young-onset CRC is approximately 44 years, with nearly three-quarters of cases occurring between 40 and 49 years. These patients are more likely to present with advanced-stage disease, often due to delays in diagnosis [3].

CRC risk is influenced by both non-modifiable factors, such as age, sex, and family history, and modifiable lifestyle factors. It is estimated that 30-50% of CRC risk is attributable to exposures including smoking, high consumption of red and processed meats, obesity, diabetes, and excessive alcohol intake [4]. If current trends persist, the incidence of CRC in individuals under 50 years of age is projected to double by 2030. While the underlying causes are not fully understood, the increase likely reflects an interplay between genetic predisposition and environmental factors. In response, the American Cancer Society (ACS) has lowered its recommended starting age for average-risk screening to 45 years, with earlier screening for those with a family history of CRC or inflammatory bowel disease (IBD) [5].

Flexible sigmoidoscopy (FSG) offers a less resource-intensive CRC screening option compared to colonoscopy, requiring no sedation and being performable by trained non-specialists [6]. Notably, most early-onset colorectal cancer (EOCRC) cases are sporadic; hereditary syndromes such as Lynch syndrome and familial adenomatous polyposis account for only about 40% of cases [7]. Compared to late-onset CRC, EOCRC is more commonly left-sided, often involves the rectum and sigmoid colon, and is more frequently diagnosed at advanced stages (III-IV). Histologically, these tumors are more likely to be poorly differentiated, with a higher prevalence of signet ring cell and mucinous subtypes [8].

Molecularly, approximately 20% of EOCRC patients carry a germline pathogenic variant in CRC-associated genes, with half involving mismatch repair (MMR) genes linked to Lynch syndrome. Adenomatous precursor lesions are less common, suggesting a more rapid carcinogenic process. CRC can be classified into three major molecular subtypes: chromosomal instability (CIN), CpG island methylator phenotype (CIMP), and microsatellite instability (MSI). MMR-deficient tumors, often MSI-high, have prognostic and therapeutic implications: they respond poorly to fluorouracil-based adjuvant chemotherapy but generally have better overall survival, and MMR-deficient metastatic tumors may benefit from immune checkpoint inhibitors [9].

Despite the rising burden of early-onset rectal cancer, data from developing countries remain limited, particularly regarding its clinical presentation, stage at diagnosis, histopathological characteristics, and associated risk factors. Understanding these patterns is essential for developing targeted screening strategies and public health interventions. This study aims to investigate the demographic, clinical, and pathological features of rectal cancer among young adults presenting to a tertiary care center, with a focus on diagnostic delays and disease stage at presentation.

## Materials and methods

This study was conducted in the Department of General Surgery, Ward II, Jinnah Postgraduate Medical Centre (JPMC), Karachi, between January 2025 and June 2025. Ethical approval was obtained prior to commencement from the College of Physicians and Surgeons Pakistan (CPSP) Research and Ethics Committee under approval number Ref No: CPSP/REU/SGR-2022-186-13709, dated December 26, 2024, and further approval was secured from the Ethical Review Committee of JPMC. Written informed consent was obtained from all participants in accordance with the principles of the Declaration of Helsinki. All patient data were anonymized and de-identified prior to inclusion in this study, ensuring full compliance with ethical standards and journal policy.

The required sample size was calculated using PASS 2020 Power Analysis and Sample Size Software (NCSS, Kaysville, Utah) [10]. Calculations assumed an expected prevalence of lymph node metastasis in young-onset rectal cancer of 57%, with a 95% confidence level, a margin of error of ±11%, and a study power of 80%, yielding a required sample size of 73 patients. As rectal cancer in young adults is relatively uncommon, non-probability purposive sampling was applied. Random sampling was not feasible, as it would not provide an adequate number of cases within the study period. Therefore, all consecutive eligible patients diagnosed during the study window were included to maximize feasibility and reflect the true burden of disease in this age group.

Inclusion criteria

Patients were between 18 and 45 years of age at the time of diagnosis, had a histologically confirmed diagnosis of rectal adenocarcinoma, presented to or received treatment at JPMC during the study period, and had complete clinical, radiological, and pathological data available. Both newly diagnosed and referred cases were considered eligible for inclusion.

Exclusion criteria

Patients were excluded if they were younger than 18 years or older than 45 years, had cancers originating outside the rectum (such as colon or anal cancers), had recurrent rectal cancer initially diagnosed outside the study period, had incomplete or missing medical records, or had received initial treatment at another institution and presented only for follow-up.

Study protocol

This cross-sectional study was conducted over six months (January to June 2025). All patients underwent colonoscopy with biopsy for histological confirmation, as well as CT and/or MRI for staging. Tumor staging was performed according to the AJCC 8th edition. Clinical history and physical examination were obtained for all patients. Medical records, imaging, and histopathology reports were reviewed to confirm diagnosis and staging. Consecutive eligible patients meeting the inclusion criteria were enrolled for analysis of presenting features and associated clinical factors.

Statistical analysis

Data were entered and analyzed using IBM SPSS Statistics for Windows, Version 26 (Released 2019; IBM Corp., Armonk, New York). Continuous variables were expressed as mean ± standard deviation (SD), while categorical variables were presented as frequencies and percentages. Analyses were descriptive in nature.

## Results

**Figure 1:**
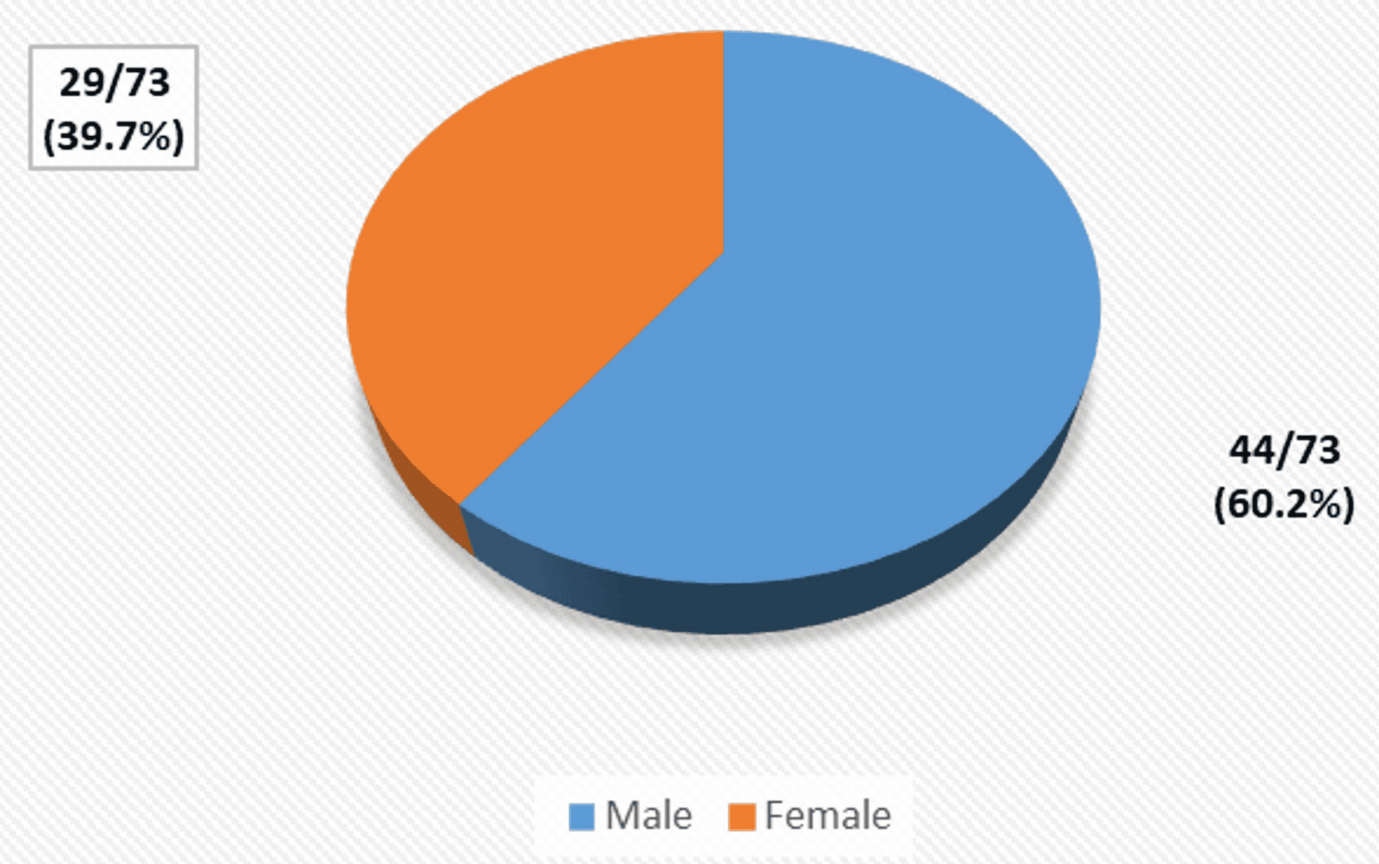
Gender distribution of young adults with rectal cancer (n = 73)

A total of 73 young adults diagnosed with rectal cancer were included in the study, comprising 44 males (60.2%) and 29 females (39.7%) (Figure 1). The mean age of the participants was 32.5 ± 7.2 years.

Presenting symptoms

The most frequently reported symptom was per rectal bleeding, present in 54 patients (74.0%). Altered bowel habits combined with lower abdominal pain were reported in 10 patients (13.7%), and fecal incontinence in 7 patients (9.6%). Two patients (2.7%) presented with acute intestinal obstruction. No patients reported tenesmus or spurious diarrhea. The distribution of presenting symptoms is summarized in Table 1.

**Table 1:** Presenting symptoms of young adults with rectal cancer (n=73). 95% confidence intervals (CI) are shown only for presenting symptoms

Symptom	Number	Percentage (%)	95% CI (Lower–Upper)
Per rectal bleed	54/73	74.0%	62.9%–82.7%
Altered bowel habits + lower abdominal pain	10/73	13.7%	7.6%–23.4%
Fecal incontinence	7/73	9.6%	4.7%–18.5%
Intestinal obstruction (emergency)	2/73	2.7%	0.8%–9.5%
Tenesmus	0/73	0.0%	0.0%–5.0%
Spurious diarrhea	0/73	0.0%	0.0%–5.0%

Risk factors

**Figure 2:**
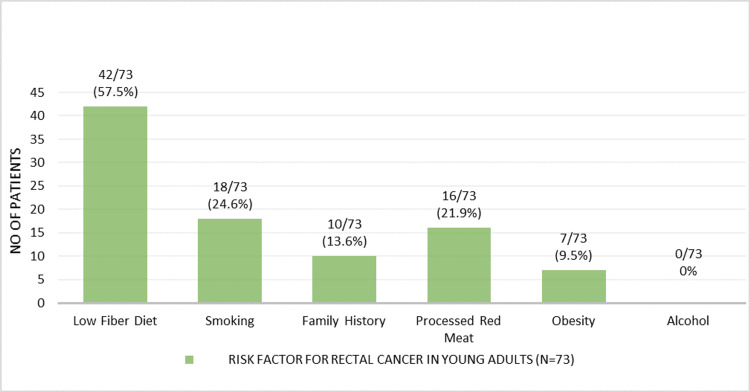
Risk factors among young adults with rectal cancer (n = 73)

A low-fiber diet was the most common risk factor, reported in 42 patients (57.5%). This was followed by smoking in 18 patients (24.6%) and consumption of processed red meat in 16 patients (21.9%). Obesity was observed in 7 patients (9.5%), and a positive family history of colorectal cancer was found in 10 patients (13.7%). None of the participants reported alcohol consumption (Figure 2).

Tumor localization

**Figure 3:**
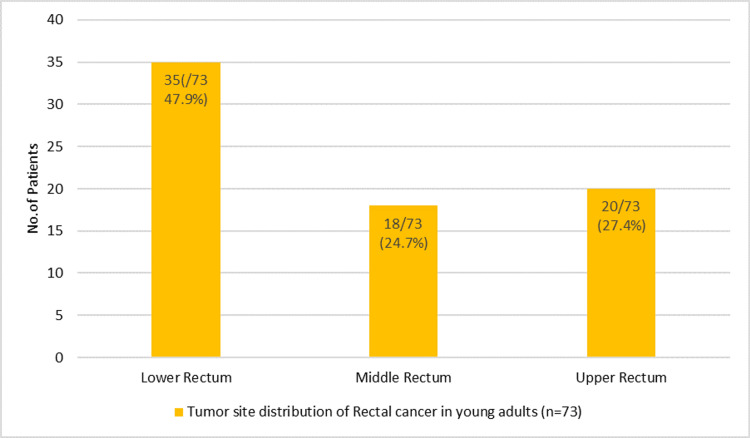
Tumor site distribution among young adults with rectal cancer (n = 73)

The lower rectum was the most frequently involved site, affecting 35 patients (47.9%). Tumors were located in the upper rectum in 20 patients (27.4%) and in the middle rectum in 18 patients (24.7%), indicating a predominance of distal tumor localization (Figure 3).

Cancer staging

**Figure 4:**
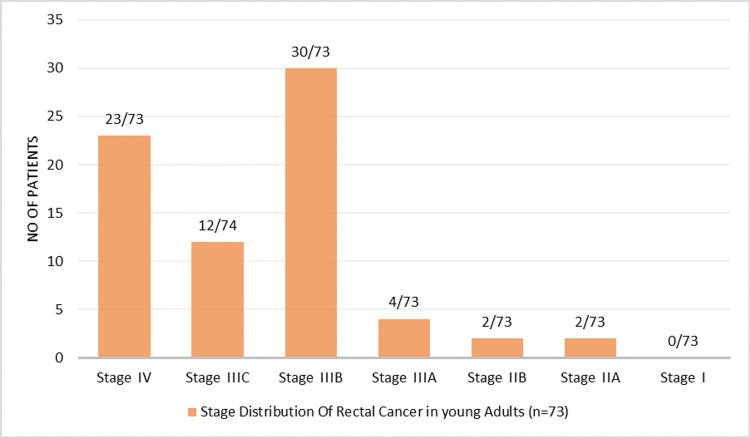
Tumor staging of young adults with rectal cancer according to AJCC 8th edition (n = 73)

Most patients presented with advanced-stage disease. Stage IIIB was the most prevalent, seen in 30 patients (41.1%), followed by Stage IV in 23 patients (31.5%) and Stage IIIC in 12 patients (16.4%). Earlier stages were uncommon: Stage IIIA in 4 patients (5.5%), Stage IIA in 2 patients (2.7%), and Stage IIB in 2 patients (2.7%). No patients were diagnosed at Stage I (Figure 4).

Histopathological findings

**Figure 5:**
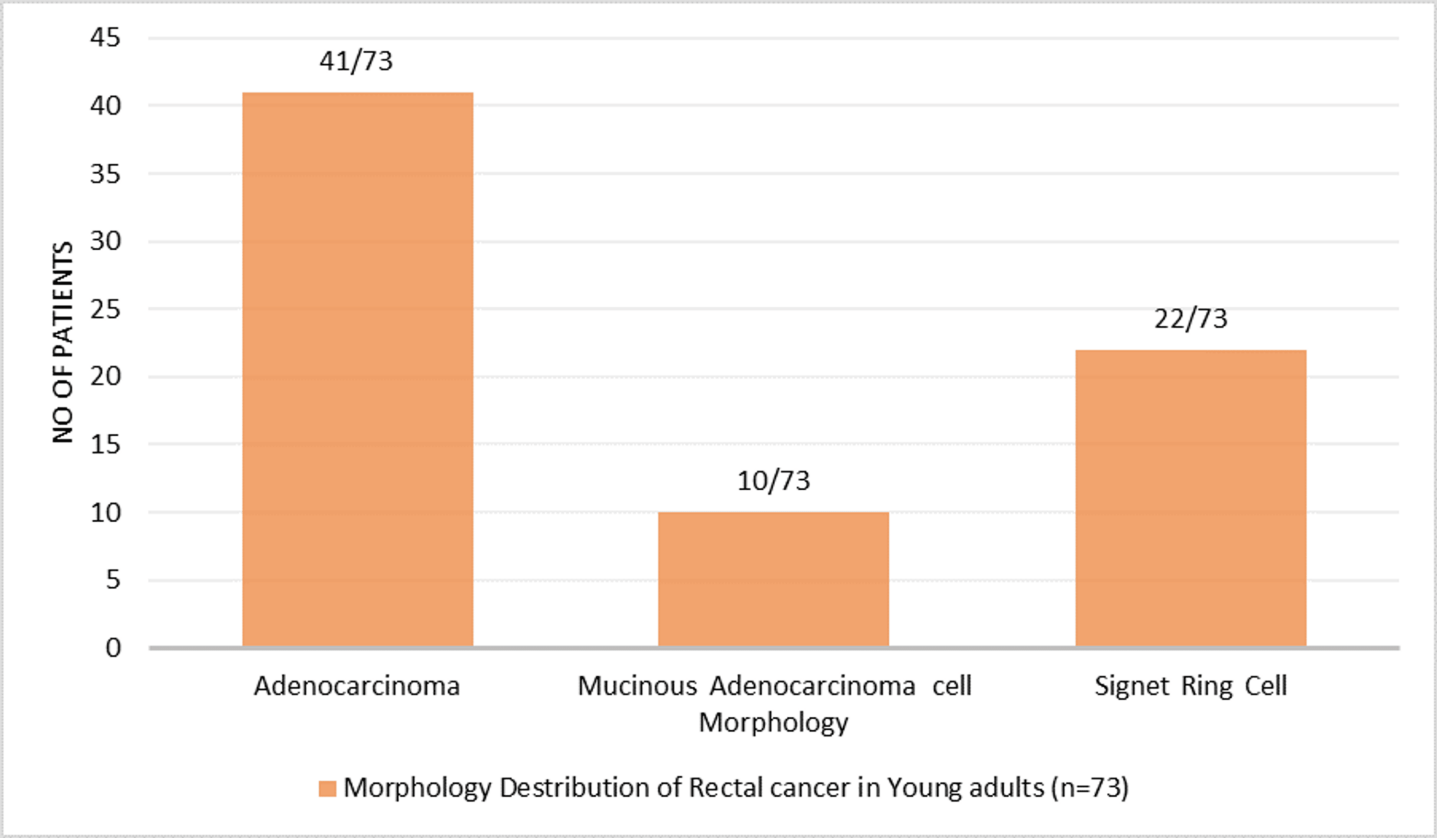
Histological subtypes of rectal cancer in young adults (n = 73)

Adenocarcinoma was the most common histological subtype, found in 41 patients (56.2%), followed by signet ring cell carcinoma in 22 patients (30.1%) and mucinous adenocarcinoma in 10 patients (13.7%) (Figure 5).

**Figure 6:**
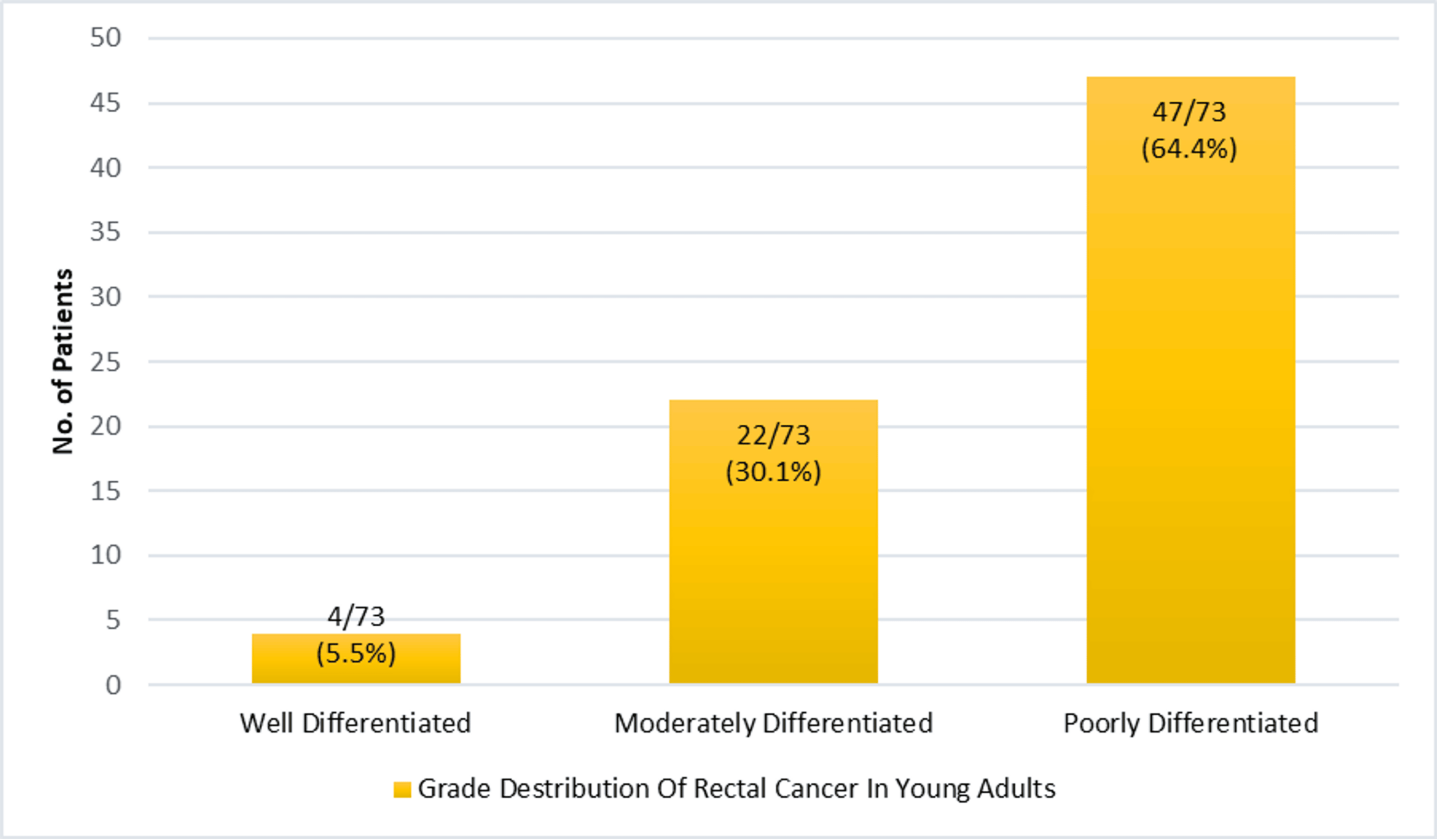
Tumor grade distribution among young adults with rectal cancer (n = 73)

Regarding tumor grade, 47 patients (64.4%) had poorly differentiated tumors, 22 patients (30.1%) had moderately differentiated adenocarcinomas, and 4 patients (5.5%) had well-differentiated tumors. These findings reflect the aggressive histopathological nature of rectal cancer in this young adult population (Figure 6).

## Discussion

In this cross-sectional study of 73 young adults diagnosed with rectal cancer, the most common presenting symptom was per rectal bleeding (74.0%), followed by altered bowel habits with lower abdominal pain (13.7%) and fecal incontinence (9.6%). A majority of patients presented with advanced-stage disease, with stage IIIB and stage IV collectively accounting for over 70% of cases. Tumor localization was predominantly in the lower rectum (47.9%), and adenocarcinoma was the most frequent histological subtype (56.2%). A significant proportion of tumors were poorly differentiated (64.4%), and signet ring cell carcinoma was observed in 30.1% of patients, indicating an aggressive disease profile in this young population. These findings underscore the concerning trend of late-stage presentation and aggressive tumor characteristics in young adults with rectal cancer. Interestingly, none of the patients in our series reported tenesmus or spurious diarrhea, symptoms occasionally described in rectal cancer. This absence may reflect differences in tumor location, disease biology, or patient-reported symptom patterns in younger adults.

The findings of our study align with global trends indicating an increasing incidence of rectal cancer among young adults, often presenting at advanced stages. In our study, 72.6% of patients were diagnosed at stage IIIB or IV, which is consistent with a multicenter European study reporting that 46% of adolescents and young adults presented with locally advanced or metastatic disease at diagnosis. Similarly, a study from India observed that 86.6% of patients under 35 years presented with advanced-stage disease [11,12]. Per rectal bleeding was the most common presenting symptom in our study (74.0%), which is comparable to findings from other studies where it was reported in approximately 68.8% of young patients [13]. This symptom is increasingly recognized as a significant red flag for early-onset colorectal cancer, underscoring the importance of timely recognition and diagnostic evaluation [14]. Furthermore, the predominance of poorly differentiated tumors (64.4%) and the high incidence of signet ring cell carcinoma (30.1%) in our study suggest a more aggressive tumor biology in young adults. These findings are supported by prior literature indicating that signet ring cell histology is more prevalent in patients under 40 years of age and is strongly associated with advanced disease and poorer outcomes [15].

The predominance of advanced-stage disease (stages IIIB and IV) in our study suggests delayed diagnosis and potentially aggressive tumor biology in young adults. Similar findings have been reported in global studies, where early-onset rectal cancer often remains unrecognized due to low clinical suspicion and diagnostic delays, despite persistent symptoms such as rectal bleeding [14]. The high frequency of poorly differentiated tumors and signet ring cell carcinoma in our study supports the hypothesis that early-onset rectal cancers may exhibit distinct and more aggressive histopathological features [11,16,17]. Signet ring cell histology, in particular, is more prevalent in younger patients and is associated with a worse prognosis and limited response to standard therapies, emphasizing the need for age-adapted treatment strategies [11,16]. Reports in the literature indicate that early-onset rectal cancer patients are more likely to present with advanced nodal disease and high-risk pathological features, including poor differentiation, mucinous or signet ring histology, and perineural invasion [18].

The predominance of advanced-stage rectal cancer in our young adults underscores the critical need for heightened clinical vigilance and earlier diagnostic interventions. Rectal bleeding, observed in 74.0% of our patients, is a significant red flag symptom associated with early-onset colorectal cancer (EOCRC). A systematic review and meta-analysis reported that hematochezia was present in 45% of EOCRC cases and was linked to a 5- to 54-fold increased risk of cancer [19]. In response to the rising incidence of EOCRC, the U.S. Preventive Services Task Force (USPSTF) updated its guidelines in 2021, recommending that colorectal cancer screening begin at age 45 for average-risk individuals [20]. However, our findings suggest that even this age threshold may be insufficient, as a significant proportion of young adults present with advanced disease before reaching 45. Therefore, clinicians should maintain a high index of suspicion for EOCRC in patients under 45 who present with persistent gastrointestinal symptoms, particularly rectal bleeding, abdominal pain, altered bowel habits, or iron deficiency anemia. Prompt referral for diagnostic colonoscopy in these cases is crucial for early detection and improved outcomes.

One of the key strengths of this study is its focus on a specific and often underrepresented population (young adults with rectal cancer) providing detailed insights into their clinical presentation, histological subtypes, and staging at diagnosis. All included cases were histologically confirmed, and complete clinical records were reviewed to ensure data accuracy. The use of a clearly defined age range (18-45 years) and a structured approach to data collection enhances the internal validity of our findings. However, this study also has several limitations. As a single-center study with a relatively small sample size, the findings may not be generalizable to broader populations or other geographic regions. Additionally, due to the cross-sectional design, we could not assess treatment outcomes or long-term survival, limiting the ability to draw conclusions about prognosis. Furthermore, risk factor data were self-reported and may be subject to recall bias. Because of the descriptive study design and limited sample size, we did not perform inferential analyses such as logistic regression or odds ratios. The analysis was therefore restricted to descriptive statistics, which provide observational insights but do not allow adjustment for confounding variables.

Our study highlights the concerning trend of late-stage presentation and aggressive tumor characteristics in young adults with rectal cancer. The predominance of symptoms such as rectal bleeding and the high incidence of poorly differentiated and signet ring cell tumors emphasize the need for greater clinical awareness and timely diagnostic evaluation in this age group. These findings support the growing call for revisiting current screening strategies and underscore the importance of recognizing early warning signs, even in individuals below traditional screening age. Further multicenter studies are warranted to expand on these findings and inform future guidelines aimed at improving early detection and outcomes in young-onset rectal cancer.

## Conclusions

This study underscores the concerning trend of rectal cancer in young adults presenting predominantly at advanced stages, often with aggressive histological features such as poorly differentiated and signet ring cell tumors. Per rectal bleeding was the most frequent presenting symptom, highlighting the importance of recognizing red flag signs even in younger patients. These findings support the need for earlier evaluation and consideration of revising current screening thresholds. Greater clinical awareness and prompt diagnostic assessment are critical for improving outcomes in early-onset rectal cancer.
